# Extensively drug resistant tuberculosis in a high income country: A report of four unrelated cases

**DOI:** 10.1186/1471-2334-8-60

**Published:** 2008-05-02

**Authors:** Stefan H Blaas, Ralf Mütterlein, Johannes Weig, Albert Neher, Bernd Salzberger, Norbert Lehn, Ludmila Naumann

**Affiliations:** 1 Department of Internal Medicine I, Regensburg University, Regensburg, Germany; 2 Center for Pneumology, Donaustauf Hospital, Donaustauf, Germany; 3 District Hospital (Bezirksklinikum), Parsberg, Germany; 4 Public Health Department, Neustadt/Waldnaab, Germany; 5 Center for Respiratory Medicine and Thoracic Surgery, Asklepios Fachkliniken, München-Gauting, Germany; 6 Institute of Medical Microbiology and Hygiene, Regensburg University, Regensburg, Germany; 7 Division of Infectious Diseases, Bavarian Health and Food Safety Authority, Oberschleissheim, Germany

## Abstract

**Background:**

Multi drug resistance *of Mycobacterium tuberculosis *(*M. tuberculosis*) remains a major threat to public health, reinforced by recent reports about the clinical course of patients infected with extensively drug resistant (XDR) strains in South Africa. There is little information about the clinical course of XDR tuberculosis patients in industrialised countries.

**Methods:**

We evaluated all isolates of *M. tuberculosis*, in which drug susceptibility testing was performed at our institution since 1997, for multi and extensive drug resistance. Clinical courses of patients infected by strains fulfilling the recently revised criteria for XDR tuberculosis were analysed.

**Results:**

Four XDR *M. tuberculosis *isolates were identified. All patients had immigrated to Germany from Russia, Georgia, and former Yugoslavia and none were infected by the human immunodeficiency virus. All patients where treated for tuberculosis for 5.5 to 15 years and for XDR tuberculosis for 1.9 to 2.5 years. They received inhospital treatment in Germany for 11 months, 4.5 years and twice for 6 years. Non-compliance was an important factor in all four patients, three patients had to be treated in Germanys only locked facility for tuberculosis treatment. One patient with XDR tuberculosis died, one patient had still open pulmonary tuberculosis at last contact and 2 patients were cured.

**Conclusion:**

Cases of XDR tuberculosis have been treated in our region for several years. Even in a high income setting, XDR tuberculosis has a tremendous impact on quality of live, outcome and the total cost. All reasonable efforts to prevent the spread of XDR tuberculosis must be made and maintained.

## Background

Tuberculosis (TB) remains one of the major causes of death from a single infectious agent worldwide. The emergence of multi drug resistance, defined as resistance to at least rifampin (RMP) and isoniazid (INH) is of great concern. Recently, reports of strains with extensively drug resistant (XDR) TB have been described and the question was raised, whether we enter the post-antibiotic era [1-4]. In October 2006 the WHO Global Task Force on XDR-TB met in Geneva, Switzerland, and approved the following revised laboratory case definition of XDR-TB: "TB showing resistance to at least rifampin and isoniazid, which is the definition of multi drug resistant (MDR) TB, in addition to any fluoroquinolone, and to at least 1 of the 3 following injectable drugs used in anti-TB treatment: capreomycin, kanamycin and amikacin" [5]. A report of 53 XDR-TB patients with a high prevalence of human immunodeficiency virus (HIV) infection from rural South Africa, showed an alarming case fatality rate of almost 100% with a median survival of 16 days [4]. Clinical data about patients with XDR TB in high income countries are scarce. We therefore reviewed all cases of XDR TB detected in our microbiology laboratory since 1997 and evaluated the clinical course and the outcome of these cases.

## Methods

Results of drug susceptibility testing (DST) for all mycobacterial isolates belonging to the mycobacterium tuberculosis complex (*M. tuberculosis*, *M. bovis*, *M. africanum*, *M. microti*, *M. canetti, M. caprae*) have been recorded in the electronic database of the Institute of Medical Microbiology and Hygiene at Regensburg University since its implementation in 1997. We identified all isolates showing resistance to INH and RMP and analysed the results of drug susceptibility testing to second line drugs to identify XDR TB strains according to the revised WHO criteria. We retrieved data pertaining clinical course, treatment, and outcome of these patients up to November 2006 from the case records and communicated with the currently treating physicians. Results of DST performed in other German laboratories have also been evaluated.

In our laboratory DST has been performed using the modified proportion method on Löwenstein-Jensen agar, broth-based radiometric (BACTEC 460 system, Becton Dickinson Diagnostic Systems, Sparks, MD).), and/or nonradiometric testing methods (BACTEC MGIT 960, Becton Dickinson Diagnostic Systems). Quality was controlled internally according to DIN 58943 (DIN standard of the German Institut for standardization) or to the manufacturer' instructions, respectively, and externally by a national quality assessment program.

For drugs used during treatment of XDR TB antituberculous drug dosing and dosing intervals is provided wheneffer different from WHO standard or when no WHO dose recommendation is provided [6]. Treatment outcome was categorised based on international standards [7]. For case number 4, the only case for which information on cost of in- and outpatient treatment was available, such data are reported.

## Results

In our Institute specimen from Regensburg University hospital, from other local hospitals in the region of Northern Bavaria, from other microbiology laboratories, and from local health authorities are analysed.

From 1997 to 2006 in 2522 strains belonging to the M. tuberculosis complex a DST was performed. 817 of these isolates were sent to us for complete or partial DST only. DST to first and second line antituberculous drugs were performed according to clinical relevance and availability of drugs at the time, when testing was performed. 2174 isolates were tested for MDR and in 470 strains at least one second line drug has been tested.

During 1997 to 2006 112 isolates from 84 patients fulfilling the criteria for multi drug resistance were identified. Isolates from 15 of 71 patients tested revealed resistance at least to one of the fluoroquinolones ofloxacin, ciprofloxacin (Cfx) or moxifloxacin (Mxf). 19 of 71 patients tested had isolates resistant to one of the i.v. second line drugs: amikacin (Am, 15 of 53 patients tested) or kanamycin (2 of 5 patients tested), and capreomycin (8 of 66 patients tested). 4 isolates fulfilled the revised criteria for XDR-TB as stated above and case records of these patients were analysed in detail. These XDR TB isolates were cultured from samples gained from 1998 to 2003.

All patients immigrated to Germany, one from former Yugoslavia and three from the Commonwealth of Independent States (alliance of 11 former Soviet Republics), where prevalence of MDR and XDR TB is particularly high (Table 1). The available information about the antituberculous treatment in the patients' country of origin is very limited.

**Table 1:** Epidemiologic factors, manifestations of tuberculosis

	Patient 1	Patient 2	Patient 3	Patient 4
Country of Origin	Georgia	Russia, Sibiria	Yugoslawia	Russia
Risc Factors for TB		smoking, alcohol abuse	smoking, alcohol abuse	smoking
HIV Status	not infected	not infected	not infected	not infected
Culture isolate	*M. tuberculosis*	*M. tuberculosis*	*M. tuberculosis*	*M. tuberculosis*
				
TB manifestation at time of immigration and at time of XDR TB diagnosis	reactivated open pulmonary tuberculosis with bilateral cavernous formations	reactivated open pulmonary tuberculosis with bilateral cavernous formations	reactivated open pulmonary tuberculosis with bilateral cavernous formations	reactivated open pulmonary tuberculosis affecting both lungs with a cavernous formation in the left upper lobe

Extrapulmonary manifestation	none	right sided Otitis media	none	none

All patients where treated for TB for 5.5 to 15 years and 1.9 to 2.5 years for XDR TB. Three received at least 4.5 years of inhospital treatment in Germany for TB (Table 2). No XDR TB patient was HIV-infected. All patients had exhibited lack of compliance, three patients had to be treated in Germanys only closed facility for tuberculosis treatment repeatedly (Table 3). One patient with XDR TB died, one patient had still open pulmonary TB at last contact and 2 patients were cured from XDR TB.

**Table 2:** Course of disease, outcome

	Patient 1	Patient 2	Patient 3	Patient 4
First time TB diagnosis	1995	1992	1985	2001
Immigration Date	August-97	July-97	March-92	March-03
Duration between TB and MDR TB diagnosis	2 years	5 years	12 years	2 years
Duration between MDR TB and XDR TB diagnosis	2 years	5 years	1.3 years	concurrent
Duration between XDR TB diagnosis and end of antituberculous drug therapy in Germany	2.4 years	1.9 years	2 years	2.5 years
Overall length of inhospital treatment in Germany	4.5 years	6 years	6 years	11 months
				
Outcome	treatment failure	cure	death	cure
Last sputum cultures	positive	negative	positive	negative
				
Radiologic signs of activity	present	not present	present	not present
				
Known transmission of XDR strain to contacts	no	no	no	no

**Table 3:** Risk factors for resistance development, drug susceptibility testing, antituberculous therapy

	Patient 1	Patient 2	Patient 3	Patient 4
Risk factors for resistance development:				
Necessity of closed ward treatment	yes	yes	yes	no
Antituberculous treatment before immigration	yes, regimen unknown	yes, including 9 months of RMP monotherapy	yes, regimen unknown	yes, regimen unknown
Drug susceptibility testing at time of Immigration:				
Resistant	INH, RMP, RFB, EMB, PZA, SM, Eto, Cs, Tsc, Clr, Cfz	INH, RMP, EMB, SM, Pto	INH, EMB	-
Intermediate	PAS	-	-	-
Susceptible	Am, CM, Ofx	PZA, Cs, Cfx	RMP, PZA, SM	INH, RMP, EMB, PZA, SM
Drug susceptibility testing at time of XDR Diagnosis:				
Resistant	INH, RMP, EMB, SM, Cm, Ofx, Eto, Pto, Cs, PAS, Tsc	INH, RMP, RFB, EMB, SM, CM, Cfx, Amx/Clv, Tsc	INH, RMP, RFB, EMB, PZA, Cm, Am, Ofx, Eto, Cs, Cfz, Tsc	INH, RMP, PZA, SM, Am, Cm, Cfx, Amx/Clv, Cfz, Tsc
Intermediate	PZA	Pto	-	-
Susceptible	Mxf, Cfz	PZA, Am, Mxf, Cs, Cfz, Lzd	SM, PAS, Clr	EMB, Mxf, Pto, Cs, PAS, Lzd
Antituberculous drugs used during therapy of XDR TB	PZA, Mxf, Pto, Cfz	PZA, Am, Mxf, Lzd	RFB, PZA, SM, Am, Cm, Ofx, Pto, Trd, PAS, Cfz, Amx/Clv, Clr, Doxycyclin	EMB, PZA, Am, Mxf, Cfx, Pto, Trd, Lzd
Non pharmacologic treatment	right upper lobectomy and atypical left upper lobectomy	right upper lobe resection 01/03 + left upper lobe and left lower lobe segmental resection 04/03		left sided pneumonectomy

XDR extensively drug resistant, TB tuberculosis, INH isoniazid, RMP rifampicin, RFB rifabutin, EMB ethambutol, PZA pyrazinamide, SM streptomycin, Km kanamycin, Am amikacin, Cm capreomycin, Cfx ciprofloxacin, Ofx ofloxacin, Lfx levofloxacin, Mfx moxifloxacin, Eto ethionamide, Pto protionamide, Cs cycloserine, Trd terizidone, PAS *p*-aminosalicylic acid, Cfz clofazimine, Amx/Clv amoxicillin/clavulanate, Clr clarithromycin, Lzd linezolid, Tsc thiosemicarbazon

*Patient 1 *is a 40 year old asylum seeker, in whom TB was diagnosed in 1995 in Georgia, where he had been treated on an in- and outpatient-basis. In August 1997 at the time of immigration to Germany he had open pulmonary TB affecting both lungs with cavernous formation bilaterally. Initial DST in Germany revealed MDR TB with additional resistance to other first and second line drugs. Due to non-compliance with the therapy he was admitted to the closed facility. Radiographic findings improved but sputum conversion was not achieved. After 10 months of treatment, a right upper lobectomy and atypical left upper lobectomy was performed, followed by a complicated postoperative course. Several DST showed a very broad spectrum of resistance without a chance of cure for any available drug regimen at that time. As the patient urged for an antituberculous drug therapy anyhow he received a treatment with INH, RMP, ethambutol (EMB), and pyrazinamide (PZA) for several months. In October 1999 a XDR strain was isolated. Despite resistance to Cfx, the strain was sensitive to Mxf so a treatment was initiated with Mxf, PZA (1500 mg/day) and clofazimine (50 mg/day). A few months later clofazimine was substituted by protionamide due to the latest DST. Protionamide was stopped because of patient refusal in 2001. In February 2002 he returned to Georgia, and we have no information about further treatment. At that time, after 4.5 years of continuous inhospital treatment, sputum conversion still had not been achieved.

*Patient 2 *is a 45 year old immigrant from Russia. He was diagnosed with TB in 1992 and received prolonged treatment, including 9 months of RMP monotherapy. He arrived in Germany in July 1997 with MDR open pulmonary TB with bilateral cavernous formations and right-sided tuberculous otitis media. Initial empirical treatment was adapted after DST, but sputum conversion was not achieved and the patient had to be admitted to our closed facility due to non-compliance the first time in April 1998. After almost three years of treatment sputum conversion was achieved with a treatment regimen adapted to the last DST including INH, EMB, Am, Mxf, *p*-aminosalicylic acid, terizidone. The patient had to be discharged due to legal requirements albeit treating physicians were unsure of the further compliance with the therapy. Shortly thereafter he was readmitted again with open pulmonary TB, probably due to non-compliance. XDR TB with resistance to Cfx but sensitivity to Mxf was diagnosed in January 2002. In January 2003 right upper lobe resection was performed and sputum conversion was again achieved under a treatment of PZA, Am (500 mg/day until March 2003), Mxf, and linezolid (600 mg twice daily, reduced to 600 mg/day in March 2003). Two months later the patient could be dismissed. In April 2003 he developed a left sided spontaneous pneumothorax which did not respond sufficiently to pleural drainage and an upper lobe and lower lobe segmental resection was performed. Oral antituberculous therapy (PZA, Mxf, and linezolid) was continued until December 2003 and there were no signs of reactivation of XDR TB since then.

*Patient 3 *was diagnosed with pulmonary TB in former Yugoslavia in 1985 at age 38. Reactivation of TB was diagnosed and treated in Italy in November 1991. The patient seeked asylum in Germany in March 1992 and received inhospital treatment for open pulmonary TB and bilateral cavernous transformation. Due to severe non-compliance he was admitted to our closed facility in October 1992, where he was treated until May 1993, when sputum conversion was achieved. He was readmitted only three months later, when sputum culture again turned positive. Further closed ward treatment was necessary, but after improvement of compliance, the patient could be discharged in May 1995. After another inhospital treatment 1996 he was again admitted for open TB in summer 1997 and came to the closed ward for the third time in October 1997, where he remained until his death in April of the year 2000. There was resistance to INH, EMB, and intermediate susceptibility to RMP in 1992 already, but it took until February 1997, when MDR TB diagnosis was definite. In May 1998 an XDR strain was isolated, only being sensitive to streptomycin, *p*-aminosalicylic acid, clarithromycin. The following drugs had been included in the different regimens before XDR TB was diagnosed: INH, RMP, EMB, PZA, streptomycin, Am, capreomycin, Cfx, ofloxacin, protionamide, *p*-aminosalicylic acid, cycloserine, clofazimine, and doxycyclin.

During the following years, strains showing various resistance patterns were isolated, but there was no curative medical or surgical option, due to extensive resistance, the patient's comorbidities and his non-compliance. He received antituberculous treatment with various agents (Table 3), but he died of progressive decompensated right heart failure.

*Patient 4 *is a 45 year old immigrant, diagnosed with TB in Russia at the end of 2001 and treated with an unknown regimen for 5 – 6 months. Since mid 2002 he again had fevers, chills, weight loss and he was admitted to hospital immediately after arrival in Germany in spring 2003. Pulmonary TB affecting both lungs with a cavernous formation in the left upper lobe was diagnosed and standard treatment initiated. Initial DST revealed *M. tuberculosis *fully sensitive to all first line drugs. After 6 months of treatment he was discharged without sputum conversion and isolated at home. DST was repeated and finally revealed an XDR TB strain. The patient was therefore again admitted to hospital with massive excretion of bacilli and empirical treatment with EMB, PZA, Am (1 g, 3 times per week), Mxf, protionamide, terizidone (750 mg/day), and linezolid (600 mg twice daily) was initiated. After 9 weeks sputum conversion was achieved, but there was no radiological improvement. As the left upper and lower lobes were involved a left-sided pneumonectomy was performed for definitive cure. While in November 2003 two specimens showed XDR strains, further isolates showed sensitivity to fluoroquinolones. Gastrointestinal drug toxicity led to withdrawal of PZA and protionamide after 4 months. Am was discontinued after 11 weeks because DST revealed resistance. EMB had also to be withdrawn due to drug toxicity. After 4.5 months developed a sensory polyneuropathy affecting primarily his feet, but because of being a mainstay of therapy, Linezolid was not discontinued but its daily dose was reduced to 600 mg once daily. Due to gastrointestinal toxicity Mxf was changed to Cfx (1000 mg once daily). After 6 months the patient was discharged and treated with Cxf, terizidone and linezolid for a total of 24 months after surgery. Directly observed therapy was organised through the local public health department and later through the family to prevent defaulting. 7 months after end of antituberculous treatment no sign of reactivation could be found. The direct medical costs in Germany were € 90.000 for inpatient treatment and € 79.000 for outpatient antituberculous drug therapy.

Systematic investigation of health care workers of all patients and of household contacts of patient 4 did not reveal transmission of XDR TB.

## Discussion

Resistance of *M. tuberculosis *is increasingly reported. Recently, data from a South African cohort of mostly HIV infected XDR TB patients with a rapid and almost uniformly fatal course of disease was presented. Of the four XDR TB patients we presented, none was HIV infected. The long and complicated history of the disease and the great impact of the non-compliance to antituberculous measures and treatment are remarkable.

Epidemiologic data published by the Centers for Disease Control and Prevention in 2006 showed a worldwide increase of XDR TB according to the previous case definition from 2000 to 2004 [3]. In contrast to this, the facts that contact investigation did not reveal transmission of XDR TB and that we have not isolated another XDR strain since 2004 suggest that XDR TB does not seem to spread rapidly in our population. All patients were either hospitalised or home isolated and under strict control of the local public health department at time of XDR TB diagnosis and none was discharged before sputum conversion.

While the incidence for tuberculosis in Germany decreased from 13.6/100,000 in 1997 to 7,3/100,000 in 2005, the proportion of MDR isolates increased from 1.3% to 2,7% [8,9]. Referral bias is the most probable reason for the higher proportion of MDR TB in our microbiology laboratory, as our laboratory receives isolates to perform DST for second line antituberculous drugs and also receives specimen from hospitals specialised in TB treatment.

A key feature in all of our patients seems to be the occurrence of mixed infection at some time point. In patient 4 at the time of XDR TB diagnosis two widely resistant strains were isolated, one showing resistance and one showing susceptibility to Cfx. The Cfx resistant strain was isolated twice within a few days, and Cfx testing was performed in Mycobacteria Growth Indicator Tube (MGIT, Becton Dickinson Diagnostic Systems, critical concentration for ciprofloxacin susceptibility: 2 mg/l), so misinterpretation seems to be unlikely. Our interpretation is that in this case there had been two strains, one XDR strain and one MDR strain still sensitive to fluoroquinolones.

In recent years several reports about heteroresistance (i.e. a mixture of resistant and susceptible subpupulations in the same culture) have shown that mixed infection is a valid phenomenon in clinical tuberculosis and is not uncommon [10-14].

Mixed infection can explain the fact, that in the patients presented the occurrence of resistance to a specific drug can not always be deduced by the initial DST and the antituberculous therapy given in the meantime. In these cases a mixed infection with an already resistant strain may have been obscured by the conventional methods of TB culture and DST.

During the long course of disease in patients 1 to 3 DST sometimes revealed isolates showing susceptibility to antituberculous drugs previously tested resistant. It has been hypothesised that the use of second line drugs leads to re-emergence of drug susceptible strains in patients with mixed infections [15].

If the mixed infection is due to unrelated strains or due to one strain with different resistance patterns remains unclear, as DNA fingerprinting or other subtyping data addressing this question is not available.

While WHO guidelines recommend the use of at least four active drugs for treatment of MDR-TB, this is often not possible for XDR-TB [6]. Individual regimens have been used in the patients presented. With only two patients cured from XDR tuberculosis outcomes are far from optimal. Cure was only achieved in patients who where operated and who had a fluoroquinolone as a therapeutic option. This underlines the impact of this drug class for treatment success in MDR and XDR TB.

Another important factor for successful treatment of resistant tuberculosis is directly observed therapy. While the current structures worked well in patient 4, poor adherence during outpatient treatment after discharge from the closed facility was an important factor for further resistance development in patients 2 and 3. This case series demonstrates that *M. tuberculosis *does not only develop extensive drug resistance in countries with suboptimal health care resources but also in our high income setting. Therefore, maintenance of structures for TB control and improvement of deficits in outpatient treatment of non-compliant patients is mandatory, even when the incidence of TB in industrialised countries remains low or even declining. As this may afford additional resources political commitment is necessary.

Our four cases also demonstrate the tremendous impact of XDR TB on quality of life and on health care resources. Three of our patients were treated in inpatient settings for 4.5 to 6 years. Not only the total length of hospital stay but also the prolonged administration of expensive and toxic second line agents, operations, non-pharmacological treatment, the use of administrative and legal resources, and last but not least the high expenses of a closed ward treatment are all contributing to the very high cost of treatment of XDR TB. Because of the extremely long course of disease, multiple treatment changes and the many health care providers involved reliable cost estimation is very difficult in patients 1 to 3. Still, in patient 4, who had by far the shortest course, treatment cost exceeded € 170,000.

Several reports have shown that MDR strains are transmitted effectively from person to person [1]. 55% of patients from the South African cohort had no previous treatment. Additionally, health care workers were affected. This demonstrates that extensive drug resistance does not diminish the contagiousness of the involved strains. Fortunately systematic contact investigation did not reveal transmission of an XDR strain from our reported patients to close contacts or health care professionals. Consequent implementation of isolation according to the recommendations of the German Central Committee against Tuberculosis, low HIV-prevalence and the medical resources of a high income country may have contributed to this finding [16].

The nightmare of patients eluding public health care measures are currently discussed in the context of a case provoking media attention and public outrage in the USA [17].

## Conclusion

In this report we present four cases with XDR TB in a high income country. Cases of XDR TB have been treated in our region for several years and fortunately it has not gained such a tremendous impact as suggested by the data from the recent report from HIV infected patients from South Africa. But even in a high income country XDR TB has a tremendous impact on quality of live, outcome and the expense of medical resources. Therefore, all reasonable efforts to prevent the development and the spread of XDR TB must be made and maintained.

## List of Abbreviations

MDR: multi drug resistant; XDR; extensively drug resistant; TB: tuberculosis; DST: drug susceptibility testing; MGIT: Mycobacteria growth indicator tube; INH: isoniazid; RMP: rifampicin; EMB: ethambutol; PZA: pyrazinamide; Am: amikacin; Cfx: ciprofloxacin; Mfx: moxifloxacin.

## Competing interests

There was no financial support for this study.

SHB has received travel grants from Actelion, Bayer Phamaceuticals, and BMS and research funding from Boehringer Ingelheim. BS has received travel grants from Bayer Pharmaceuticals and Pfizer and for participations in advisory boards from Pfizer. NL has received lecture fees from Pfizer, AstraZeneca, and Bayer Pharmaceuticals and grants from Pfizer and Bayer Phamaceuticals.

RM, JW, AN, and LN have no competing interests.

## Authors' contributions

SHB, BS, NL, and LN devised the study protocol. SHB analysed the results of DST as well as the clinical data and drafted the manuscript. RM, JW, AN provided information about the clinical course of the patients and helped to draft the manuscript. BS helped to draft the manuscript. NL provided the results of the DST from the electronic database. LN performed most of the resistance testing and helped to draft the manuscript. All authors read and approved the final manuscript.

## Pre-publication history

The pre-publication history for this paper can be accessed here:


